# Eyelid Eccrine Poroma: A Case Report and Review of Literature

**DOI:** 10.7759/cureus.8906

**Published:** 2020-06-29

**Authors:** Enrique Mencía-Gutiérrez, Carmen Navarro-Perea, Esperanza Gutiérrez-Díaz, María Cámara-Jurado, Álvaro Bengoa-González

**Affiliations:** 1 Ophthalmology, 12 De Octubre Hospital, Complutense University, Madrid, ESP; 2 Pathology, 12 De Octubre Hospital, Complutense University, Madrid, ESP

**Keywords:** eyelid, eccrine poroma, tumor, benign, diferential diagnosis, excision, epidermal, swet duct unit, porocarcinoma, acrosyringium

## Abstract

Poroma is a rare benign tumor of the epidermal sweat duct unit with predilection for the head and neck. Only six cases with eyelid location have been described in the literature (PubMed). A 34-year-old male presented with a single tumor on the left upper eyelid. It was skin-colored, nodular, solid, tender with some telangiectatic vessels, and showed no ulcerated lesion. Clinical diagnosis was basal cell carcinoma. This type of lesion can mimic a malignancy. Complete excisional biopsy revealed features consistent with eccrine poroma. After three year of follow up, no recurrence was observed. The authors reviewed all the cases reported in the literature and made a summary comparing them.

## Introduction

Poromas are common benign tumors of the epidermal sweat duct unit. First described by Pinkus et al. in 1956 [1], they are subclassified as eccrine poroma, hidroacanthoma simplex, poroid hidradenoma and dermal duct tumors [2]. Poromas usually occur on the palms and soles. They rarely occur on the eyelid and should be considered in the differential diagnosis of eyelid tumors, particularly with a suspected basal cell carcinoma. This case presentation discusses the clinical and microscopic features of poroma. No apocrine differentiation has ever been observed in this location. They usually appear as solitary papule or nodule in middle-aged to elderly men and women. Our patient, in this case, is the youngest (only 34 years) among all patients that are described in the published literature. Complete primary excision and subsequent microscopic examination are advocated. The possible malignant transformation is described in the literature as porocarcinoma. We report a case of eyelid eccrine poroma and compare this to the only six cases previously described (PubMed) [3-8].

## Case presentation

**Figure 1:**
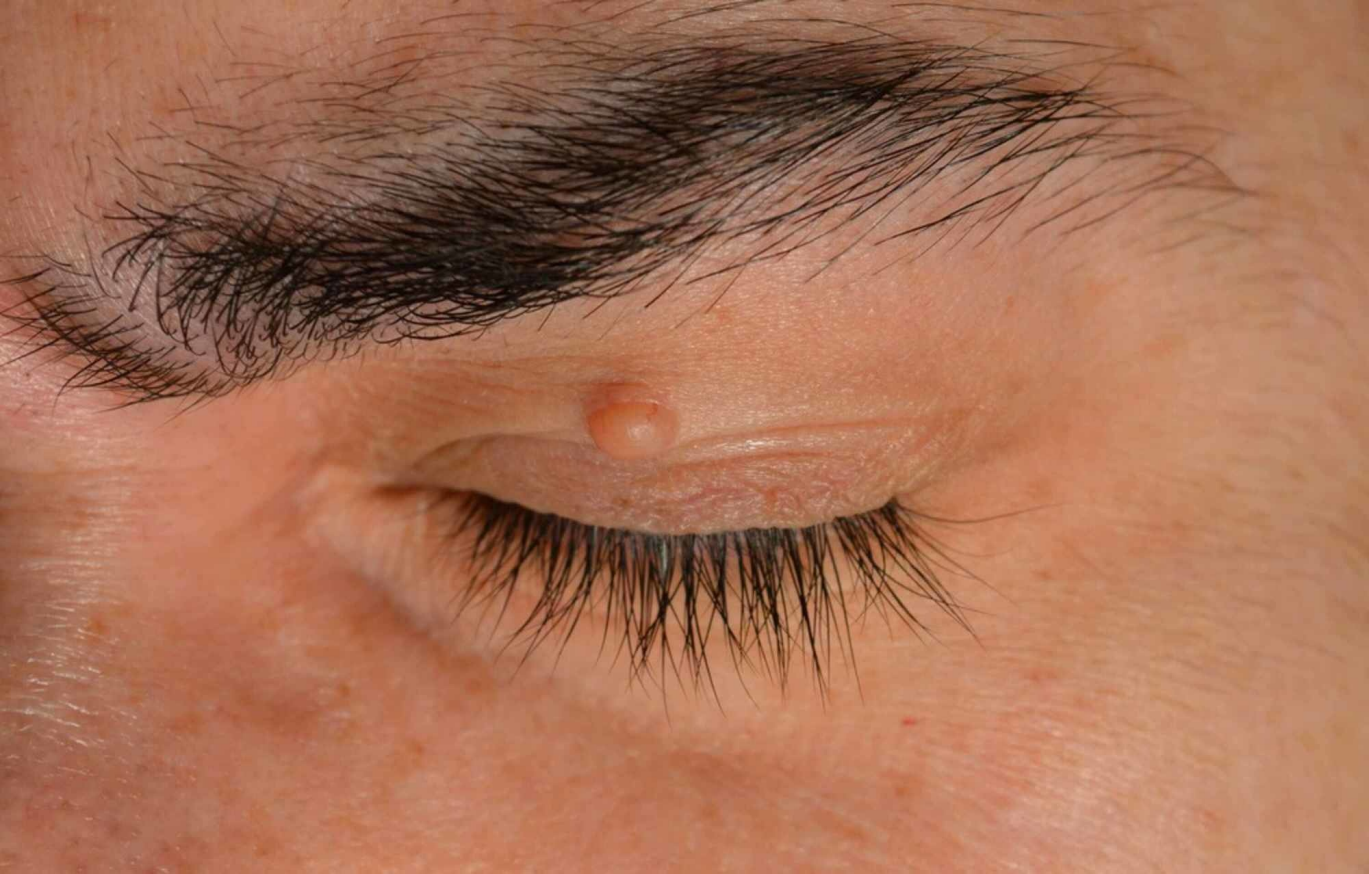
Skin-colored nodule, smooth-surface on the left upper eyelid, 6 x 3 mm in size.

**Figure 2:**
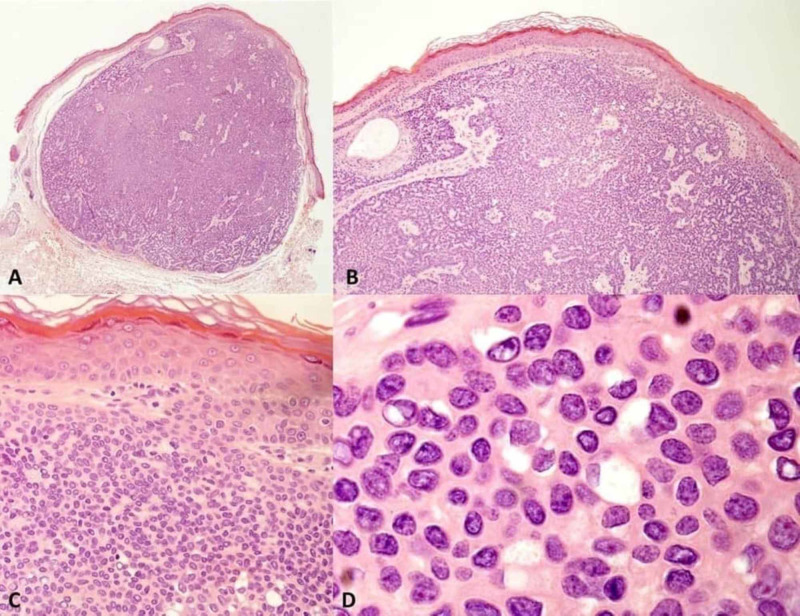
A, Well-circumscribed, totally excised tumor with lobulated growth pattern, with ticked cords of tumor cells that surround a vascular and fibrotic stroma (hematoxylin-eosin stain (H&E), 4x). B, The tumor is connected to the overlying epidermis and extends into the papillary and reticular epidermis. Circumscribed lobulated growth pattern is observed, with thicket cords of tumor cells that surround a vascular a fibrotic stroma, (H&E, 10x). C, The tumor is composed of small cuboidal keratinocytes with small bland nuclei (small nuclei showing a uniform chromatin) (H&E, 40x). D, Small ductal lumina with ducts are often found (H&E, 100x).

A 34-year-old male presented with a single slow-growing asymptomatic tumor. It had appeared one year before on the upper left eyelid, located in the middle third of the lid, and coextending into crease and eyebrow. The lesion measured 6 x 3 mm in size. It was skin-colored, nodular-regular, tender, solid, not ulcerated, with some telangiectasia vessels on the surface of the tumor. Clinical diagnosis was basal cell carcinoma (Figure 1). The patient had no other lesions on the skin. The lesion was excised under local anesthesia and with macroscopic free margins of 3 mm. Pathologic study demonstrated a tumor with a nodular silhouette, well-demarcated on macroscopic image (Figure 2A). Microscopic examination with hematoxylin-eosin stain showed the tumor connected to overlying epidermis and extending into the papillary and reticular dermis (Figure 2B). Circumscribed lobulated growth pattern was observed, with thickened cords of tumor cells that surrounded a vascular and fibrotic stroma (Figure 2C). It was composed of small cuboidal keratinocytes with small bland nuclei (small nuclei showing uniform chromatin). Small ductal luminal (ducts) were often found (Figure 2D). Surgical margin was free. According to this description, the diagnosis was a poroma. There has been no local recurrence in a follow-up of over three years. The aesthetic result was very good.

## Discussion

Poroma is a relatively little known skin tumor with multilineage differentiation. It is a benign cutaneous neoplasm related to the acrosyringium, and included in a group of benign adnexal neoplasm with ductal differentiation [1]. They appear preferentially on the face, palms, or soles and rarely outside of these areas [2]. Nonetheless, eccrine poroma may be found in any skin area bearing sweat glands [9]. On the eyelids, only six cases of poroma have been reported (Table1) [3-8]. Nine of the reported porocarcinomas state that they may arise de novo, or develop from a pre-existing poroma through degenerative progression [10-12].

**Table 1:** Summary of cases of poroma involving the eyelids. M=male, F=female, RC=right canthus, RL=right lower, LU=left upper, RLE=right lower eyelid, BCC=basal cell carcinoma

	Age (years)/ Sex	Location/ eyelid	Size (mm) (length x height)	Color/type	Follow-up (months) Previous/postsurgery	Margin involved	Other skin lesions	Clinic diagnosis	Histologic description
Fujita M et al. 1986 [3]	70/F	RC/inner	10x10x9	Red-dome-shaped/Keratinous mass	2/12	No	Syringocystadenoma papilliferum	Keratinous mass	Folliculare eccrine poroma
Vu PP et al. 2001 [4]	71/M	RL/inner	15x13	Peau d’orange/Nodule	96/36	No	BCC (preauricular)	BCC	Eccrine poroma
Chen CC et al. 2006 [5]	76/M	RU/lateral	3x3	Red/Papule	24/No data	No	No data	Soft fibroma	Eccrine poroma
Rabady DZ et al. 2008 [6]	71/F	RL/lateral	5x5	Flesh-red/Papule	No data/12	No	Nevus RLE (multiple facial)	No data	Intraepidermal eccrine poroma
Iwasaki J et al. 2008 [7]	91/M	RL/medial	4x5	Reddish-pink/Nodule	12/No data	Yes	No data	BCC	Sebaceous differentiation eccrine poroma
Ahuja S et al. 2019 [8]	70/M	RU/medial	30x10	Pigmented/Pea-sized	72/6	No	Eccrine poroma	No data	Intraepidermal eccrine poroma
Current	35/M	LU/medial	6x3	Skin/Nodule	12/36	No	No	BCC	Eccrine poroma

Adnexal tumors are classified based on two characteristics: benign versus malignant (the most important issue) and line of differentiation. This means determining which normal cutaneous structure the lesion most closely resembles (hair follicle, apocrine/eccrine gland, sebaceous gland). There is controversy about this subclassification and the nomenclature is still confusing. Fortunately, this is not relevant because the biological potential in all the different histological subtypes of poromas is the same. Although poromas have traditionally been thought to originate from the eccrine sweat gland, there have been cases classified as “apocrine” or “apocrine-folliculosebaceous” as well. Our case could be considered of an “eccrine type” because we cannot identify any feature suggestive of apocrine differentiation like columnar cells with holocrine decapitation secretions or “snouts” or folliculosebaceous lineage (sebaceous-like component). Carcinoembryonic antigen immunostaining can identify the presence of both eccrine and apocrine ducts and thus help in the identification. If the poroma displays more tubular foci lined by columnar cells with holocrine secretions, this may be highly suggestive of an apocrine etiology. It is thought that a poroma with sebaceous differentiation is most likely to be of folliculosebaceous-apocrine lineage [11]. Sometimes, tumor cells can show a squamoid appearance, or be clear or pigmented. Mitotic figures may be present and can be numerous in traumatized lesions [13].

Poromas affecting the head and neck are more typically of apocrine origin. On the eyelid, all cases described were eccrine [3-8]. Rare cases associated with radiation therapy and pregnancy have been described [14]. They are rarely multiple [15].

In poromas, the mean age at diagnosis was 68.3 years. Males showed a much higher incidence than females with a ratio of 15:1. In those reported on the eyelid, the mean age was 69.1, and the proportion male to female 5:2. Our case is the youngest one at only 34 years and was a male. All the lesions were single, and varied in mean size from 0.7 to 1 cm in diameter; and in the eyelid, the range was 0.4 to 2 cm in diameter. This case is within the mean, at 0.6 cm. Most of the lesions were asymptomatic as in the presented case. Previous evolution time was 36 months, and in the cases reported in the eyelid, it was the same. In our case, it was only one year, a possible sign of malignancy. The color of lesions varies from red to brown; in our case, it was skin-colored. None of the tumors on the eyelid had a correct preoperative diagnosis [5]. The main misdiagnosis was a basal cell carcinoma.

Selected differential diagnosis must be made with benign eccrine sweat gland tumors such as hidradenoma, syringoma, chondroid syringoma among others, as well as with irritated seborrheic keratosis. Differential diagnosis must also be made with a malignant eccrine sweat gland tumor as porocarcinoma and with a basal cell carcinoma, squamous cell carcinoma, sebaceous gland carcinoma (it can be difficult to differentiate clinically) and in general with other lesions of the eyelid and periocular skin.

Poroma lacks horn pseudocysts of seborrheic keratosis and clefts, peripheral palisading, and atypia nuclear of basal cell carcinoma. In contrast, a careful search for small ductal luminal or cuticles, sometimes better highlighted with immunostains for carcinoembryonic antigen or stains for periodic acid-Schiff, leads to a correct diagnosis of poroma [11].

Poroma also needs to be distinguished from its malignant counterpart, the porocarcinoma, which may arise from it or simulate features of poroma. Porocarcinomas will usually show an infiltrative growth pattern and marked atypia. It is important to know that we can find extensive necrosis, mitosis, and also epithelial atypia in benign poromas when they are mechanically irritated [11].

## Conclusions

To date, there have been only six cases of eyelid poroma reported in the literature (PubMed). It is a benign neoplasm of the eccrine gland with ductal differentiation. The etiology is not clear. They are thought to differentiate towards (or originate from) the distal part of the sweat duct. They were formerly considered eccrine but may also present with apocrine differentiation (in our case, apocrine differentiation was not found). The definitive treatment of eccrine poroma consists of complete surgical excision with clear margins to avoid local recurrence. It is therefore important to consider it in the differential diagnosis of any eyelid tumor. The main problem is mistaking it with a carcinoma and performing an unnecessary extensive resection.
